# Clinical Significance in Oral Cavity Squamous Cell Carcinoma of Pathogenic Somatic Mitochondrial Mutations

**DOI:** 10.1371/journal.pone.0065578

**Published:** 2013-06-14

**Authors:** Chih-Hsiung Lai, Shiang-Fu Huang, Chun-Ta Liao, I-How Chen, Hung-Ming Wang, Ling-Ling Hsieh

**Affiliations:** 1 Graduate Institute of Biomedical Sciences, College of Medicine, Chang Gung University, Tao-Yuan, Taiwan; 2 Department of Otorhinolaryngology, Head and Neck Surgery, Chang Gung Memorial Hospital, Tao-Yuan, Taiwan; 3 Division of Hematology/Oncology, Department of Internal Medicine, Chang Gung Memorial Hospital, Tao-Yuan, Taiwan; 4 Department of Public Health, College of Medicine, Chang Gung University, Tao-Yuan, Taiwan; University of Porto, Portugal

## Abstract

Somatic mutations affecting the mitochondrial DNA (mtDNA) have been frequently observed in human cancers and proposed as important oncological biomarkers. However, the clinical significance of mtDNA mutations in cancer remains unclear. This study was therefore performed to explore the possible clinical use in assessing oral squamous cell carcinoma (OSCC) of pathogenic mtDNA mutations. The entire mitochondrial genome of 300 OSCC with their matched control DNAs was screened by direct sequencing and criteria were set to define a pathogenic somatic mutation. The patients' *TP53* R72P genotypes were determined by polymerase chain reaction-restriction fragment length polymorphism. The relationships between pathogenic somatic mutations, clinicopathogical features, *TP53* R72P genotype and clinical prognosis were analyzed. Overall, 645 somatic mtDNA mutations were identified and 91 of these mutations were defined as pathogenic. About one quarter (74/300) of the OSCC tumor samples contained pathogenic mutations. Individuals with the *TP53* R allele had a higher frequency of pathogenic somatic mutation than those with the PP genotype. Kaplan-Meier analysis indicated that *TP53* R allele patients with pathogenic somatic mutations demonstrated a significant association with a poorer disease-free survival than other individuals (HR = 1.71; 95% CI, 1.15–2.57; *p* = 0.009) and this phenomenon still existed after adjusting for mtDNA haplogroup, tumor stage with treatment regimens, differentiation and age at diagnosis (HR = 1.59; 95% CI, 1.06–2.40; *p* = 0.03). Subgroup analyses showed that this phenomenon was limited to patients who received adjuvant radiotherapy/chemo-radiotherapy after surgery. The results strongly indicated that pathogenic mtDNA mutations are a potential prognostic marker for OSCCs. Furthermore, functional mitochondria may play an active role in cancer development and the patient's response to radiotherapy/chemo-radiotherapy.

## Introduction

Oral cancer is currently a major global health issue. In Taiwan, oral cancer has been the fourth most common cancer in men since 2006 [1]. According to the cancer registry report from the Department of Health of the Executive Yuan, the annual age-adjusted incidence for oral cancer in males has shown a 1.55-fold increase in the past decade [2]. Since prognosis remains poor despite progress in the disease's management [3], there is a need to gather clues that will help to unravel the underlying mechanisms involved in the biological behaviors of this disease.

A rich and complex body of knowledge gained from cancer research has revealed that cancer is a disease that involves dynamic changes in both the nuclear and mitochondrial genome. Observations in human cancers and animal models have provided concrete evidence indicating that tumor development proceeds via a succession of cellular DNA alterations, each conferring a growth advantage and that these changes lead to the progressive conversion of normal cells into cancer cells [4]. On the other hand, there is still a debate as to whether mitochondrial DNA (mtDNA) mutations are actually a cause of tumor progression due to the lack of distinct biochemical and molecular pathways toward tumorigenicity [5]. Nonetheless, the multiple hallmarks of cancer cells, including limitless proliferative potential, insensitivity to anti-growth signals, impaired apoptosis, enhanced anabolism and decreased autophagy, have been linked to mitochondrial dysfunction [6]. Mitochondrial DNA (mtDNA) is a double-stranded circular genome of 16.5 kb in length. mtDNA has a higher mutation rate than nuclear DNA [7], [8] and mutations are commonly found in different types of human tumors including head and neck cancers [9], [10]. These findings suggest a potential role for mtDNA mutations in tumor development. Recently, Larman et al. [11] found high-impact somatic non-synonymous mtDNA mutations in tumor tissue samples from 226 individuals with five types of cancer and this further strengthens the evidence that mtDNA mutations might confer selective growth advantages early in oncogenesis. However, the clinical use of these mtDNA mutations was not addressed.

The tumor suppressor protein p53 is well known as a central factor for the maintenance of nuclear genome stability and for the suppression of cancer. Recently, increasing evidence has shown that p53 also plays an important role in regulating mtDNA repair, replication and recombination [12]. The *TP53* gene contains a nonsynonymous single nucleotide polymorphism (SNP) that results in either an arginine (R) or proline (P) at position 72 of the p53 protein. The two resulting variants are different with respect to modulating apoptosis, to translocating to the mitochondria, to being degraded by the proteasome and to binding to MDM2 [13]. Since p53 participation in mtDNA repair has been identified in a variety of systems [14], [15], [16], [17], it is possible that the *TP53* R72P (rs1042522) polymorphism contributes to the differences related to mtDNA mutations in human cancers. This study was therefore performed to explore the landscape of mtDNA mutations by sequencing the complete mitochondrial genome of 300 oral squamous cell carcinoma (OSCC) patients, to assess the relationship between the *TP53* R72P polymorphism and mtDNA mutations, and to examine the possible clinical use of pathogenic mtDNA mutations. Our results suggest for the first time that pathogenic mtDNA mutations are potential prognostic markers for OSCC and *TP53* R72P polymorphism was associated with pathogenic mtDNA mutations.

## Materials and Methods

### Patients and sample specimens

This study was approved by the Institutional Review Board, Chang Gung Memorial Hospital. A series of 300 male OSCC patients treated at Chang Gung Memorial Hospital, Linkou, during the period from March 1999 to October 2005 were recruited for participation in this study [18]. All cases were histologically confirmed and gave written informed consent for participation before surgery. Clinical histological parameters were carefully reviewed and scored. The protocols for recommending adjuvant radiotherapy/chemo-radiotherapy, as well as the follow-up schedule, were carried out according to the hospital guidelines of care [18]. All cases were followed up until death or until June 2011 and the median follow-up time was 69.0 months. Information on the patient's history of cigarette smoking, alcohol drinking, and areca quid (AQ) chewing were obtained by uniform interview by a well-trained technician using a questionnaire. Buffy coat cells from 10 ml of venous blood, tumor tissue and nontumor matching tissue were collected, frozen in liquid nitrogen and stored at −80°C until DNA extraction. Genomic DNA was purified as previously described [19].

### PCR direct sequencing of entire mitochondrial genome and genotyping of the *p53* gene

Both forward and reverse sequencing reactions were carried out using the same primers as the PCR amplification according to manufacturer's instructions and analyzed on an ABI3130 Avent Genetic Analyzer (Applied Biosystems, Foster City, CA) as described previously [18]. The DNAs from tumor tissue and matched nontumor tissue or buffy coat cells of the same patient were analyzed. The direct comparison of the entire mtDNA sequences of the tumor tissue relative to the matched buffy coat cells or nontumor tissue was adopted to clarify mtDNA somatic mutations. The *TP53* R72P patient genotype was determined using DNAs from the buffy coat cells by polymerase chain reaction-restriction fragment length polymorphism (PCR-RFLP) as described previously [20]. All analysis was conducted in a manner that was blinded to the clinical data.

### Criteria for defining a pathogenic mutation

In order to set criteria to define a pathogenic mutation, the mitochondrial genome was classified into four functional regions (D-loop, tRNAs, rRNAs, and protein-coding genes). It has been suggested that big deletions in D-loop may have some effect on mitochondrial function, although the precise consequence of D-loop mutations are still unknown. Accordingly, only unusual alterations (such as 50 bp deletion) in the D-loop that were present in a tumor were defined as possible pathogenic mutations. Pathogenicity of tRNA and rRNA mutations was characterized because they may change evolutionarily conserved nucleotides and thus affect RNA secondary structure, which can have a significant effect on function [21]. For the 13 protein-coding genes, we used two *in silico* algorithms (the PolyPhen-2 algorithm [22] and the SIFT algorithm [23]), two amino acid substitution scoring matrices (the Grantham [24] and BLOSUM 62 matrix), the evolutionary conservation of each amino acid [25], and the MutPred score [26] to predict the putative effect of each nonsynonymous mutation on protein function. A pathogenic mutation was designated when at least three methods indicated that there ought to be a deleterious effect (Table S1).

### Statistical analysis

Associations between mtDNA mutations, *TP53* R72P polymorphism and clinical features were analyzed using χ^2^ test. The survival curves were constructed by the Kaplan-Meier method and compared using the log-rank test. Multivariate survival analysis was performed using a Cox proportional hazards model. All statistical analysis was performed in SPSS version 13.0 software and significant differences were considered when two-sided *p*<0.05.

## Results

### The somatic mutation spectrum of the mitochondrial genome of Taiwanese OSCCs

To define the pattern and frequency of mtDNA mutations, the entire mitochondrial genome of 300 primary OSCC samples were sequenced. The sequencing results for the tumor DNA were compared with those obtained from either matching peripheral white blood cells (n = 236) or normal adjacent tissues (n = 64, for those patients whose DNAs from peripheral white blood cells were not available). The distribution of mtDNA haplotype was not different between the DNA from peripheral white blood cells (n = 236) and normal adjacent tissues (n = 64) (*p* = 0.93). Overall, 240 of the OSCC tumor tissue samples (80%) had one or more somatic mutations (78 OSCC tissues had one mutation, 78 OSCC tissues had two mutations and 84 OSCC tissues had at least 3 mutations). In total, 85% (203/240) of somatic mutation in the OSCC cases occurred in the D-loop region of the mitochondrial genome, 47.1% (113/240) occurred in the protein coding genes, 13.8% (33/240) in occurred in the rRNAs and 8.3% (20/240) occurred in the tRNAs.

**Figure 1:**
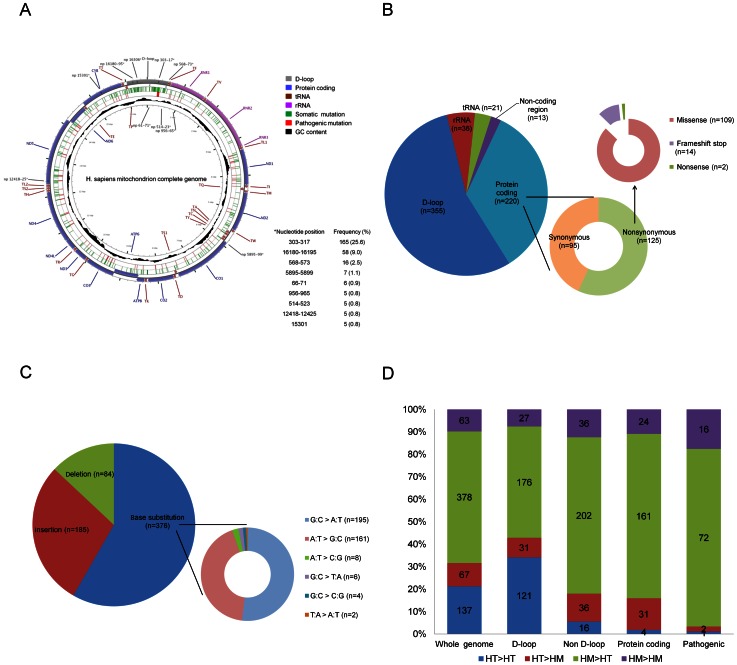
Summary of the somatic mtDNA mutations found in 300 oral cavity squamous cell carcinomas. (A) The spectrum of somatic mutations in the whole mtDNA genome (produced using CGView). Tracked on the circularized mtDNA genome, from outer-most to inner-most, the information indicated is regions of the mtDNA genome, somatic mutations show as green ticks (nucleotide position of mutation hotspots were marked with *), pathogenic somatic mutations show as pink ticks, and GC content. (B) Distribution of the 645 somatic mutations across the various different regions of the mtDNA genome. (C) Mutation types of the 645 somatic mutations. (D) Distribution of somatic mutation shifting pattern by region across the mtDNA genome and the defined pathogenic mutations. HT: heteroplasmy; HM: homoplasmy.

Overall, there were 645 somatic mutations at 329 unique nucleotide positions that were identified (Figure 1A). Among the 329 unique nucleotide positions, 181 (71 synonymous and 110 nonsynonymous) nucleotide positions were in protein-coding genes. Among the 110 nonsynonymous mutation positions, 39 had not been reported previously (Table S2). Three hundred and fifty-five (55.0%) of 645 somatic mutations were located in the D-loop and 290 (45.0%) were located in non-D-loop regions (Figure 1B). Among the 220 mtDNA mutations in protein-coding genes, 56.3% (125/220) were nonsynonymous mutations, of which missense mutations formed 87.2% (109/125), frameshift stop mutations formed 11.2% (14/125), and nonsense mutations formed 1.6% (2/125). The relative mutation frequency (mutations/nucleotides) for the D-loop region was 15.6-fold higher than for the other regions (355/1121 versus 290/15448). The most frequent mutations were in the D310 polycytidine stretch region (C-tract, 12–18 cytidine bases interrupted by a thymidine base at position 310), which is where 165 (25.6%, 165/645) mutations were found (Figure 1A).

The most common mutation type was base substitution (58.3%, 376/645), and G to A or C to T transition mutations accounted for the most mutations (51.9%, 195/376) followed by A to G or T to C mutations (42.8%, 161/376) (Figure 1C). The most common type of tumor shifting was homoplasmy>heteroplasmy (two or more populations of mtDNA in a single individual; 378/645 (58.6%); Figure 1D).

### Nature of the somatic mtDNA variants and mutation frequency

Among the ten somatic mtDNA hot mutation sites (Figure 1A), six were known to be mono- nucleotide/di-nucleotide repeat length polymorphic sites, namely nucleotide position 303–317 (D310), 514–523, 568–573, 956–965, 5895–5899 and 16180–16195. Since it has been reported that the majority of D310 mutations are observed when the number of cytosine is more than 7 [27] and when the patient has D310 heteroplasmy in the lymphocyte mtDNA [28], the relationships between the germline mono- nucleotide/di-nucleotide repeat length, heteroplasmy status and mutation frequency was analyzed. Indeed, the mutation frequency at these six mono- nucleotide/di-nucleotide repeat length polymorphic sites was found to be strongly associated with the repeat length and the heteroplasmy status (Table 1).

**Table 1:** The relationships between the number of the repeat length, status of homoplasmy/heteroplasmy and mutation frequency.

mtDNA length polymorphic site	Nucleotide position	Sequence motif in rCRS	Variant type	N	Insertion/deletion mutation frequency N (%)	OR (95% CI)	*p*
Hypervariable region 1 (HVR 1)	303–317	C_7_TC_5_GC	≤C_7_				
			Homoplasmy	112	17 (15.2)	1	
			≥C_8_				
			Homoplasmy	99	70 (70.7)	13.5 (6.6–28.1)	<0.001
			Heteroplasmy	89	74 (83.1)	27.6 (12.2–63.8)	<0.001
CA repeat	514–523	(CA)_5_	≤ (CA)_5_				
			Homoplasmy	298	3 (1.0)	1	
			≥ (CA)_6_				
			Homoplasmy	1	1 (100.0)	∞ (1.9-∞)	0.027
			Heteroplasmy	1	1(100.0)	∞ (1.9-∞)	0.027
C stretch 1	568–573	C_6_	C_6_				
			Homoplasmy	285	3 (1.1)	1	
			≥C_7_				
			Heteroplasmy	15	13 (86.7)	611.0 (76.4–7179.1)	<0.001
12S rRNA	956–965	C_5_TC_4_	C_5_				
			Homoplasmy	292	0 (0)	1	
			≥C_7_				
			Homoplasmy	1	0 (0)	-	-
			Heteroplasmy	7	5 (71.4)	∞ (69.2-∞)	<0.001
C stretch 2	5895–5899	C_5_	C_5_				
			Homoplasmy	291	2 (0.7)	1	
			≥C_6_				
			Homoplasmy	5	1 (20.0)	36.1 (1.1–759.6)	<0.001
			Heteroplasmy	4	4 (100.0)	∞ (50.6-∞)	<0.001
Hypervariable region 2 (HVR 2)	16180–16195	A_4_ C_5_TC_4_AT	≤C_5_				
			Homoplasmy	181	2 (1.1)	1	
			≥C_7_				
			Homoplasmy	9	4 (44.4)	71.6 (8.3–780.6)	<0.001
			Heteroplasmy	110	37 (33.6)	45.4 (10.3–279.7)	<0.001

### Characteristics of the somatic mtDNA mutations that affect the mitochondrial respiratory complexes

The effect of somatic mtDNA mutation on each gene and the various different respiratory complexes encoded by the mitochondrial genome were evaluated in terms of mutation load. Mutation load was calculated as total number of nucleotides altered/total number of nucleotides ×100 and only coding nonsynonymous mtDNA mutations were considered. The total and pathogenic mutation load the mitochondrial respiratory complexes were 1.10% and 0.76%, respectively. The mutation load was not significantly different among the various respiratory complexes (Table 2).

**Table 2:** Mutation load in mitochondria-encoded genes of the respiratory complex I, III, IV and V.

Gene name	Genome size (bp)^a^	Number of altered nucleotides^b^		Mutation load (%, (95% confidence interval))	
		All	Pathogenic	All	Pathogenic
Complex I					
ND1	956	15	14	1.57 (0.96–2.57)	1.46 (0.88–2.44)
ND2	1042	8	3	0.77 (0.40–1.51)	0.29 (0.10–0.84)
ND3	346	5	3	1.45 (0.64–3.33)	0.87 (0.32–2.51)
ND4	1378	14	11	1.02 (0.61–1.70)	0.80 (0.45–1.42)
ND4L	297	1	1	0.34 (0.08–1.86)	0.34 (0.08–1.86)
ND5	1812	22	16	1.21 (0.81–1.83)	0.88 (0.55–1.43)
ND6	525	6	6	1.14 (0.54–2.47)	1.14 (0.54–2.47)
Total	6356	71	54	1.12 (0.89–1.40)	0.85 (0.65–1.11)
Complex III					
CYTB	1141	13	6	1.14 (0.67–1.94)	0.53 (0.25–1.14)
Complex IV					
COXI	1542	11	9	0.71 (0.40–1.27)	0.58 (0.31–1.10)
COXII	684	6	3	0.88 (0.41–1.90)	0.44 (0.16–1.27)
COXIII	784	12	11	1.53 (0.88–2.66)	1.40 (0.79–2.49)
Total	3010	29	23	0.96 (0.67–1.38)	0.76 (0.51–1.14)
Complex V					
ATP6	681	9	3	1.32 (0.71–2.49)	0.44 (0.16–1.28)
ATP8	207	3	1	1.45 (0.53–4.16)	0.48 (0.12–2.65)
Total	888	12	4	1.35 (0.78–2.35)	0.45 (0.18–1.15)

a MITOMAP database (http://www.mitomap.org/MITOMAP).

b Only nonsynonymous coding mtDNA mutations were considered.

### Association between mtDNA haplogroup, clinicopathological features and somatic mtDNA mutations

Among the recruited 300 patients with OSCC, 240 patients carried somatic mutations in the mtDNA genome. As summarized in Table 3, no significant association was found between mtDNA haplogroup, the clinicopathological features and the somatic mtDNA mutations for the whole mitochondrial genome. However, regional mtDNA mutation analysis showed that tumor differentiation was significantly correlated with the mtDNA mutation in tRNA regions (*p* = 0.02). In addition, alcohol drinking and cigarette smoking was also found to be slightly associated the mtDNA mutation in tRNA regions (*p* = 0.05 and 0.07, respectively).

**Table 3:** Association between clinicopathological parameters, mtDNA haplogroup, *TP53* R72P polymorphism and somatic mtDNA mutation frequency.

Variables	Whole genome	*p*	D-loop region	*p*	Non-D-loop region	*p*	Protein-coding genes	*p*	rRNA	*p*	tRNA	*p*	Pathogenic	*p*
	N (%)		N (%)		N (%)		N (%)		N (%)		N (%)		N (%)	
Age		0.23		0.65		0.17		0.09		0.82		0.16		0.46
<51 (n = 149)	115 (77.2)		99 (66.4)		67 (45.0)		49 (32.9)		17 (11.4)		13 (8.7)		34 (22.8)	
≥51 (n = 151)	125 (82.8)		104 (68.9)		80 (53.0)		64 (42.4)		16 (10.6)		7 (4.6)		40 (26.5)	
Cigarette smoking		0.34		0.81		0.45		0.73		0.45		0.07		0.57
Yes (n = 242)	191 (78.9)		163 (67.4)		116 (47.9)		90 (37.2)		25 (10.3)		13 (5.4)		58 (24.0)	
No (n = 58)	49 (84.5)		40 (69.0)		31 (53.4)		23 (39.7)		8 (13.8)		7 (12.1)		16 (27.6)	
Alcohol drinking		0.91		0.72		0.65		0.93		0.67		0.05		0.46
Yes (n = 147)	118 (80.3)		98 (66.7)		74 (50.3)		55 (37.4)		15 (10.2)		14 (9.5)		39 (26.5)	
No (n = 153)	122 (79.7)		105 (68.6)		73 (47.7)		58 (37.9)		18 (11.8)		6 (3.9)		35 (22.9)	
Areca quid chewing		0.42		0.73		0.90		0.56		0.14		0.66		0.68
Yes (n = 244)	193 (79.1)		164 (67.2)		120 (49.2)		90 (36.9)		30 (12.3)		17 (7.0)		59 (24.2)	
No (n = 56)	47 (83.9)		39 (69.6)		27 (48.2)		23 (41.1)		3 (5.4)		3 (5.4)		15 (26.8)	
Tumor site		1.00		0.94		0.27		0.33		0.61		1.00		0.25
Tongue (n = 104)	83 (79.8)		69 (66.3)		48 (46.2)		36 (34.6)		13 (12.5)		7 (6.7)		24 (23.1)	
Bucca (n = 105)	84 (80.0)		72 (68.3)		48 (45.7)		37 (35.2)		9 (8.6)		7 (6.7)		22 (21.0)	
Others (n = 91)	73 (80.2)		62 (68.1)		51 (56.0)		40 (44.0)		11 (12.1)		6 (6.6)		28 (30.8)	
Tumor differentiation		0.59		0.37		0.32		0.42		0.22		0.02		0.87
Well (n = 116)	91 (78.4)		82 (70.7)		61 (52.6)		47 (40.5)		16 (13.8)		3 (2.6)		28 (24.1)	
Moderate/poor (n = 184)	149 (81.0)		121 (65.8)		86 (46.7)		66 (35.9)		17 (9.2)		17 (9.2)		46 (25.0)	
Tumor stage		0.45		0.99		0.77		0.49		0.80		0.80		0.37
I/II (n = 127)	99 (78.0)		86 (67.7)		61 (48.0)		45 (35.4)		12 (9.4)		9 (7.1)		28 (22.0)	
III/IV (n = 173)	141 (81.5)		117 (67.6)		86 (49.7)		68 (39.3)		21 (12.1)		11 (6.4)		46 (26.6)	
*TP53* R72P genotype		0.80		0.18		0.01		0.001		0.33		0.77		0.01
RR/RP (n = 204)	164 (80.4)		133 (65.2)		110 (53.9)		90 (44.1)		20 (9.8)		13 (6.4)		59 (28.9)	
PP (n = 96)	76 (79.2)		70 (72.9)		37 (38.5)		23 (24.0)		13 (13.5)		7 (7.3)		15 (15.6)	
mtDNA haplogroup		0.57		0.22		0.25		0.56		0.11		0.05		0.32
CZD (n = 91)	71 (78.0)		57 (62.6)		40 (44.0)		32 (35.2)		6 (6.6)		10 (11.0)		19 (20.9)	
Others (n = 209)	169 (80.9)		146 (69.9)		107 (51.2)		81 (38.8)		27 (12.9)		10 (4.8)		55 (26.3)	

### Association between patient *TP53* R72P genotype and somatic mtDNA mutations

Individuals with the *TP53* R allele showed a significantly higher frequency of somatic mtDNA mutations in non-D-loop regions, mainly in protein-coding genes, than individuals with the PP genotype (Table 3). As would be expected from this result, individuals with the *TP53* R allele also showed a higher frequency of pathogenic somatic mtDNA mutations than those with the PP genotype (Table 3).

### Pathogenic somatic mtDNA mutations and the survival of OSCC patients

**Figure 2:**
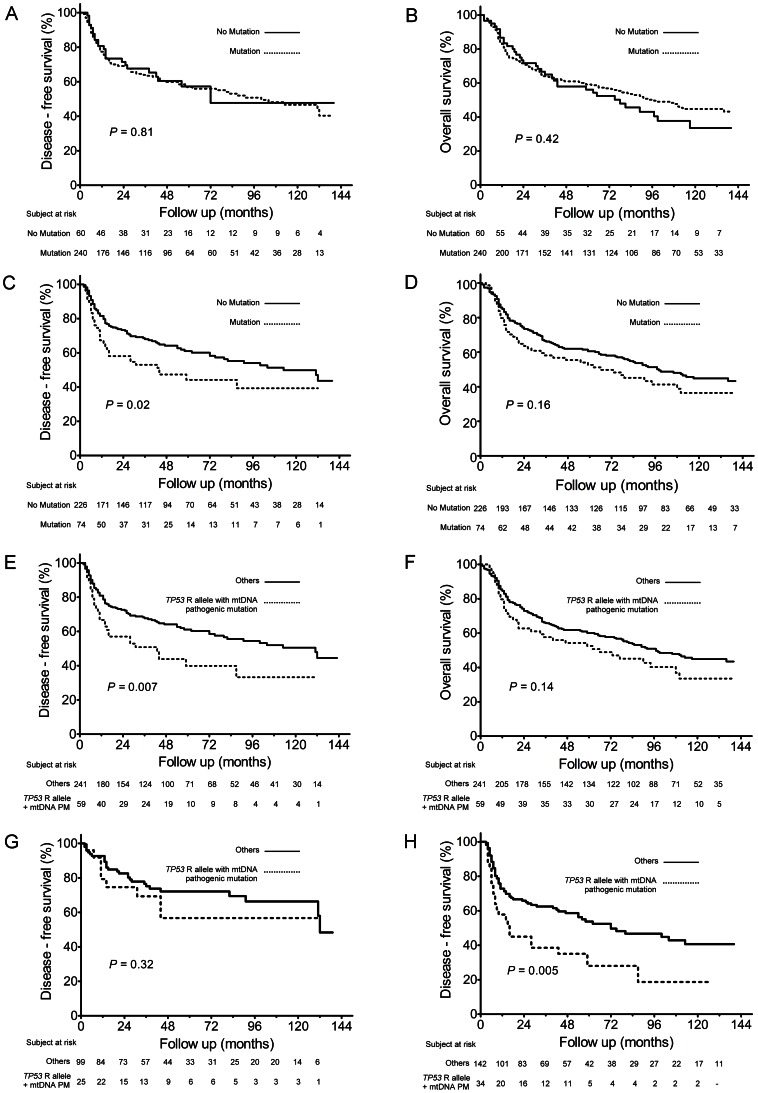
Kaplan-Meier survival curves for the OSCC patients. **(A) Disease-free survival (DFS) based on the mtDNA somatic mutation status.** (B) Overall survival (OS) based on mtDNA somatic mutation status. (C) DFS based on mtDNA pathogenic mutation status. (D) OS based on mtDNA pathogenic mutation status. (E) DFS based *TP53* R allele with mtDNA pathogenic mutation (PM) status. (F) OS based on *TP53* R allele with mtDNA pathogenic mutation (PM) status. (G) DFS based on *TP53* R allele with mtDNA pathogenic mutation (PM) status in the subgroup of patients who received surgery only. (H) DFS based on *TP53* R allele with mtDNA pathogenic mutation (PM) status in the subgroup of patients who received radiotherapy/chemo-radiotherapy after surgery.

Kaplan-Meier plot analysis indicated that somatic mtDNA mutations as a whole (including non-pathogenic mutations) were not associated with either disease-free survival (DFS) (Figure 2A) or overall survival (OS) (Figure 2B). On the other hand, pathogenic somatic mtDNA mutations were significantly associated with poor DFS (hazard ratio (HR) = 1.57; 95% confidence interval (CI), 1.07–2.30; *p* = 0.02; Figure 2C, Table 4), while, there was slight effect on OS (*p* = 0.16, Figure 2D). Interestingly, the *TP53* R allele patients with pathogenic somatic mtDNA mutations demonstrated a significant association with a poorer DFS than other patients (HR = 1.71; 95% CI, 1.15–2.57; *p* = 0.009; Figure 2E, Table 4) and this phenomenon still existed after adjusting for mtDNA haplogroup, tumor stage with treatment regimens, differentiation and age at diagnosis (HR = 1.59; 95% CI, 1.06–2.40; p = 0.03; Table 4). In addition, subgroup analyses showed this phenomenon was limited to patients who received adjuvant radiotherapy/chemo-radiotherapy after surgery (Figure 2G, 2H). On the other hand, the *TP53* R allele with pathogenic somatic mtDNA mutations was not associated with OS (Figure 2F).

**Table 4:** Cox regression analysis of disease-free survival in 300 OSCC patients.

			Univariate			Multivariate		
Parameters	5-Year (%)	10-Year (%)	HR	95% CI	*p*	HR	95% CI	*p*
Age (years old)								
<51	53.7	47.5	1			1		
≥51	60.3	45.0	0.84	0.59–1.19	0.32	0.90	0.63–1.29	0.56
Differentiation								
Well	61.5	38.0	1			1		
Moderate/poor	54.2	52.8	1.09	0.76–1.56	0.63	1.07	0.75–1.54	0.71
Tumor stage with treatment regimens								
I/II with surgery only	73.1	66.7	1			1		
I/II with surgery plus RT/CT	61.3	46.3	1.50	0.80–2.84	0.21	1.57	0.83–2.97	0.17
III/IV with surgery only	54.6	54.6	1.69	0.83–3.42	0.15	1.66	0.82–3.37	0.16
III/IV with surgery plus RT/CT	45.9	33.6	2.45	1.57–3.84	<0.001	2.38	1.52–3.73	<0.001
mtDNA Pathogenic mutation								
No	61.0	49.8	1					
Yes	44.2	39.3	1.57	1.07–2.30	0.02			
*TP53* R72P genotype								
PP	62.0	53.9	1					
RR/RP	54.5	43.6	1.34	0.90–1.98	0.15			
*TP53* R72P genotype/pathogenic mtDNA mutation								
Others	61.1	50.5	1			1		
R allele/Yes	39.9	33.2	1.71	1.15–2.57	0.009	1.59	1.06–2.40	0.03
mtDNA haplogroup								
Others	49.9	39.8	1			1		
CZD	72.7	62.7	0.52	0.34–0.80	0.003	0.56	0.36–0.86	0.009

Abbreviations: HR, hazard ratio; CI, confidence interval; RT, radiotherapy; CT, chemotherapy.

## Discussion

The sequencing of the entire mitochondrial genome allowed the construction of a mutation landscape. We have completed sequencing mitochondrial genome of 300 pairs of primary OSCC using matched blood or adjacent normal tissue and demonstrated that mono-/di-nucleotide repeat regions within the D-loop and non-coding region were the hotspots for somatic mutations in OSCC because 39.7% (265/645) of OSCC sample mtDNA was found to be mutated in this region. Most of these mutations were insertions or deletions. Our study has confirmed that D310 was the most frequent mutation site [27], [28]. Furthermore, our study also supported the idea that the number of mono- nucleotide/di-nucleotide repeats and the presence of heteroplasmy were indeed associated with the incidence of these mutations (Table 1) [27], [28]. Therefore, the distribution of repeats number and the status of heteroplasmy in the study population may explain the discrepancies in incidence of mutations, especially in D-loop, among published results [27], [29].

Overall, 80% of OSCC tumor tissues contained mitochondrial mutations. The distribution of mutation type may be able to provide some insights into mutation-causing mechanisms. The majority of the base substitution mutations were either C to T (51.9%) or A to G (42.8%) transitions, a spectrum characteristic of oxidative DNA damage [30]. To investigate the possibility that certain portions of the mitochondrial genome might be associated with cancer development, we assessed the mutation load in each region, gene or functional complex. We found that the D-loop region had a 15.6-fold higher mutation load than the other regions. It is likely that this is due to an increased rate of replicative errors in two well-known mono-nucleotide polymorphism repeats located in the D-loop region as stated above, although we can not rule out this observation is also possibly a result of differences in mutational selection in non-coding regions. Electron transport chain (ETC) complex I has commonly been considered to be a mutational hotspot in tumor mtDNA relative to other complexes [31]; however, the mutation load was not significantly different among respiratory complexes in the present study. Therefore, the more frequent identification of mutations in complex I may simply be a consequence of its greater size of this gene rather than an effect of differential selection [32].

The human mtDNA sequence is highly variable. It is necessary to define functionally important somatic mutations that may be deleterious or pathogenic in terms of tumor progression. Numerous computational algorithms using genetic, biochemical, and computational methods have been developed for predicting the pathogenicity of nonsynonymous mutations [26]. Although these methods are useful in practice, their accuracy remains a concern. Therefore, we applied a battery of six methods to define pathogenic mutations as described in the “Materials and Methods” and found that 23.6% of OSCCs carried nonsynonymous pathogenic mutations, which is closed to the results of a study of five cancers (26% to 28%) defined using Mutation Assessor by Larman et al. [11] Using this approach, the concordance with the predictions obtained from the newly developed Mutation Assessor [33] was 85.3%.

p53 has a well-known tumor suppressor ability associated with DNA repair, cell-cycle arrest, apoptosis, and senescence, which preserve genomic stability and prevent tumor formation [34]. Lately, other roles for p53 in autophagy, glucose metabolism, miRNA maturation and the control of mtDNA replication/repair have been described [35], [36], [37]. More recently, the discovery of new target genes for p53 has revealed unexpected functions for this tumor suppressor in the regulation of glucose metabolism and in oxidative stress; these have brought the fields of mitochondrial and nuclear genome together in relation to cancer biology [35], [38]. Functional interactions between p53 and mitochondria has been demonstrated, including cooperation with the mitochondrial BER repair system [15], [16], functional interaction with mitochondrial proteins [17], [37], [39], and the maintenance of mitochondrial genome stability [40]. Accordingly, a link between p53 and mitochondria in relation to tumorigenesis seems to highly likely.

The *TP53* gene has a number of allelic variants, among which the R72P polymorphism is of significant interest. The R allele of the polymorphism increases p53-induced apoptosis, whereas the P allele affects cell cycle arrest at G1 phase and DNA-repair capacity [41], [42]. We have reported that OSCC patients with the R allele show a significantly higher frequency of *TP53* mutation than those with PP genotype [20]. In the present study, we further found that OSCC patients with the R allele show a significantly higher frequency of pathogenic somatic mtDNA mutation than those with the PP genotype. Recently, Zhou et al. [9] demonstrated that there was a positive correlation between *TP53* mutations and mtDNA mutations in head and neck squamous cell cancers. Tumor tissues from 160 of the 300 presently studied patients had previously undergone mutation analysis of exons 4–10 [20]. We found that *TP53* R allele cooperated with the *TP53* mutation and enhanced significantly the risk of pathogenic somatic mtDNA mutations (Table S3). Thus, the present observations provide useful insights into the functional differences associated with the *TP53* R72P polymorphism as well as mutations of *TP53* in terms of guardianship of the mtDNA genome.

In the present study, there was no association between the presence of pathogenic somatic mtDNA mutations and clinicopathological parameters including tumor stage (Table 3). This observation is similar to the results of the recent study by Larman et al. [11] that targeted five cancers and further supports the hypothesis that damaging mtDNA mutations confer a selective advantage early in oncogenesis. Interestingly, our results suggest for the first time that pathogenic mtDNA mutations are potential prognostic markers for OSCC. In a multivariate analysis, the hazard ratio of DFS among *TP53* R allele patients with pathogenic somatic mtDNA mutations was significantly higher than among other patients and this was found to be independent of other prognostic factors. Subgroup analyses further showed that this phenomenon was limited to patients who received adjuvant therapy after surgery. These results suggest that *TP53* R allele in patients with pathogenic somatic mtDNA mutations might induce resistance to radiotherapy/chemo-radiotherapy among OSCCs. Although no experimental data supports the role of *TP53* R allele in relation to pathogenic somatic mtDNA mutations and resistance to radiotherapy/chemo-radiotherapy, p53 R is known to localize to the mitochondria more efficiently than p53 P and mitochondrial overexpression of p53 might lead to decreased mitochondrial functionality [43], [44]. Pre-existing mitochondrial dysfunction may be able to protect cells from γ-irradiation induced death by suppressing *TP53* expression/function [45]. Furthermore, it is noteworthy that 80% of the pathogenic mutations in the present study were heteroplasmic (Figure 1D). It has been shown that cells carrying a heteroplasmic rather than a homoplasmic mtDNA mutation exhibit resistance to oxidative stress-induced cell death through AKT activation [46], [47]. Taken together, the results from the present study and others support the idea that functional mitochondria may play an active role in cancer development and responses to radiotherapy/chemo-radiotherapy.

## Supporting Information

Table S1 **Predicting putative effect of nonsynonymous somatic mutations on protein function.**
(DOC)

Table S2 **Summary of 181 mutation positions in protein-coding genes.**
(DOC)

Table S3 **Combined effect of TP53 R72P polymorphism and mutation on pathogenic somatic mtDNA mutation.**
(DOC)
