# Supplementary material for: Clinical Significance in Oral Cavity Squamous Cell Carcinoma of Pathogenic Somatic Mitochondrial Mutations
Source: PLoS One. 2013 Jun 14;8(6):e65578. doi: 10.1371/journal.pone.0065578 (PMC3683038; doi:10.1371/journal.pone.0065578)
Supplement: Table S1 — Predicting putative effect of nonsynonymous somatic mutations on protein function. (DOC) [file pone.0065578.s001.doc]

Table S1. Predicting putative effect of nonsynonymous somatic mutations on protein function.

| Locus/np | SAP |  |  | Prediction methods*^a^* |  |  |  | Total scores showing deleterious effect/ Pathogenicity |
| --- | --- | --- | --- | --- | --- | --- | --- | --- |
|  |  | Grantham | EC_EU | BLOSUM62 | SIFT | PolyPhen | MutPred |  |
| Complex I |  |  |  |  |  |  |  |  |
| MT-ND1 |  |  |  |  |  |  |  |  |
| 3337 | V11M | 21 | EU | 1 | 0.32 (T) | 0.000 (B) | 0.599 | 1/No |
| 3380 | R25Q | 43 | EC | 1 | 0.00 (D) | 0.996 (ProD) | 0.915 | 4/Yes |
| 3394 | Y30H | 83 | EC | 2 | 0.04 (D) | 0.012 (B) | 0.729 | 4/Yes |
| 3398 | M31T | 81 | EC | -1 | 0.06 (T) | 0.001 (B) | 0.769 | 4/Yes |
| 3460 | A52T | 58 | EC | -1 | 0.00 (D) | 0.987 (ProD) | 0.765 | 6/Yes |
| 3599 | L98P | 98 | EU | -3 | 0.00 (D) | 0.919 (ProD) | 0.688 | 5/Yes |
| 3733 | E143K | 56 | EC | 1 | 0.00 (D) | 0.528 (Pos D) | 0.928 | 5/Yes |
| 3781 | S159P | 74 | EC | -1 | 0.00 (D) | 0.984 (Pro D) | 0.736 | 6/Yes |
| 3980 | M225T | 81 | EU | -1 | 0.00 (D) | 0.703 (Pos D) | 0.595 | 5/Yes |
| 4066 | L254F | 22 | EC | 0 | 0.05 (D) | 0.330 (Pos D) | 0.59 | 4/Yes |
| 4106 | T267I | 89 | EC | -2 | 0.03 (D) | 0.919 (ProD) | 0.573 | 6/Yes |
| 4148 | R281H | 29 | EC | 0 | 0.00 (D) | 0.895 (Pro D) | 0.833 | 3/Yes |
| 4232 | T309I | 89 | EC | -2 | 0.13 (T) | 0.000 (B) | 0.581 | 4/Yes |
| MT-ND2 |  |  |  |  |  |  |  |  |
| 4491 | V8I | 29 | EU | 3 | 1.00 (T) | 0.000 (B) | 0.371 | 0/No |
| 4665 | A66T | 58 | EC | -1 | 0.00 (D) | 0.999 (Pro D) | 0.742 | 6/Yes |
| 5007 | A180T | 58 | EC | -1 | 0.00 (D) | 0.993 (Pro D) | 0.765 | 6/Yes |
| 5104 | T212I | 89 | EC | -2 | 0.00 (D) | 0.987 (Pro D) | 0.72 | 6/Yes |
| 5140 | S224N | 46 | EU | 1 | 0.58 (T) | 0.025 (B) | 0.563 | 1/No |
| 5178 | M237L | 15 | EU | 2 | 0.55 (T) | 0.346 (Pos D) | 0.099 | 1/No |
| 5460 | T331A | 58 | EU | -1 | 0.23 (T) | 0.000 (B) | 0.193 | 2/No |
| MT-ND3 |  |  |  |  |  |  |  |  |
| 10068 | A4T | 58 | EU | -1 | 0.15 (T) | 0.003 (B) | 0.346 | 2/No |
| 10158 | S34P | 74 | EU | -1 | 0.03 (D) | 0.048 (B) | 0.342 | 3/Yes |
| 10243 | F62S | 155 | EC | -2 | 0.00 (D) | 0.970 (Pro D) | 0.776 | 6/Yes |
| 10371 | E105K | 56 | EC | 1 | 0.00 (D) | 0.975 (Pro D) | 0.874 | 5/Yes |
| 10398 | T114A | 58 | EU | -1 | 0.36 (T) | 0.000 (B) | 0.007 | 2/No |
| MT-ND4L |  |  |  |  |  |  |  |  |
| 10579 | M37T | 81 | EU | -1 | 0.00 (D) | 0.000 (B) | 0.569 | 4/Yes |
| MT-ND4 |  |  |  |  |  |  |  |  |
| 11040 | L94P | 98 | EC | -3 | 0.00 (D) | 0.906 (Pro D) | 0.761 | 6/Yes |
| 11055 | L99P | 98 | EC | -3 | 0.00 (D) | 0.906 (Pro D) | 0.865 | 6/Yes |
| 11148 | L130S | 145 | EC | -2 | 0.00 (D) | 0.981 (Pro D) | 0.666 | 6/Yes |
| 11394 | L212P | 98 | EC | -3 | 0.00 (D) | 0.440 (Pos D) | 0.746 | 6/Yes |
| 11406 | L216P | 98 | EC | -3 | 0.00 (D) | 0.981 (Pro D) | 0.89 | 6/Yes |
| 11432 | I225V | 29 | EC | 1 | 0.00 (D) | 0.408 (Pos D) | 0.702 | 4/Yes |
| 11711 | A318T | 58 | EC | -1 | 0.00 (D) | 0.927 (Pro D) | 0.747 | 6/Yes |
| 11982 | L408P | 98 | EC | -3 | 0.00 (D) | 0.952 (Pro D) | 0.724 | 6/Yes |
| 12008 | G417S | 56 | EU | 0 | 0.00 (D) | 0.981 (Pro D) | 0.698 | 4/Yes |
| 12026 | I423V | 29 | EU | 1 | 0.06 (T) | 0.002 (B) | 0.331 | 0/No |
| 12030 | N424S | 46 | EU | 1 | 0.37 (T) | 0.000 (B) | 0.259 | 0/No |
| 12128 | F457L | 22 | EU | 0 | 0.86 (T) | 0.000 (B) | 0.442 | 0/No |
| MT-ND5 |  |  |  |  |  |  |  |  |
| 12358 | T8A | 58 | EU | -1 | 0.48 (T) | Unknow | 0.277 | 2/No |
| 12398 | I21T | 89 | EU | -2 | 0.61 (T) | Unknow | 0.418 | 2/No |
| 12508 | D58N | 23 | EU | 1 | 0.28 (T) | 0.002 (B) | 0.583 | 1/No |
| 12547 | T71A | 58 | EU | -1 | 0.04 (D) | 0.012 (B) | 0.273 | 3/Yes |
| 12572 | S79N | 46 | EC | 1 | 0.06 (T) | 0.736 (Pos D) | 0.683 | 3/Yes |
| 12590 | F85S | 155 | EC | -2 | 0.00 (D) | 0.858 (Pro D) | 0.799 | 6/Yes |
| 12736 | A134T | 58 | EC | -1 | 0.00 (D) | 0.876 (Pro D) | 0.765 | 6/Yes |
| 12748 | F138L | 22 | EC | 0 | 0.07 (T) | 0.002 (B) | 0.591 | 2/No |
| 13154 | I273T | 89 | EU | -2 | 0.12 (T) | 0.185 (B) | 0.597 | 3/Yes |
| 13406 | R357E | 54 | EC | 0 | 0.00 (D) | 0.736 (Pos D) | 0.887 | 5/Yes |
| 13610 | R425Q | 43 | EC | 1 | 0.00 (D) | 0.954 (Pro D) | 0.834 | 4/Yes |
| 13753 | S473P | 74 | EU | -1 | 1.00 (T) | 0.000 (B) | 0.285 | 2/No |
| 13904 | S523P | 74 | EC | -1 | 0.00 (D) | 0.858 (Pro D) | 0.906 | 6/Yes |
| 13928 | S531T | 46 | EU | 1 | 1.00 (T) | 0.205 (Pos D) | 0.313 | 1/No |
| 14036 | S567L | 145 | EC | -2 | 0.01 (D) | 0.736 (Pos D) | 0.628 | 6/Yes |
| 14045 | Q570R | 43 | EC | 1 | 0.01 (D) | 0.328 (Pos D) | 0.662 | 4/Yes |
| MT-ND6 |  |  |  |  |  |  |  |  |
| 14226 | R150C | 180 | EU | -3 | 0.06 (T) | 0.158 (B) | 0.601 | 3/Yes |
| 14208 | A156T | 58 | EU | -1 | 0.5 1(T) | 0.003 (B) | 0.513 | 3/Yes |
| 14169 | E169K | 56 | EC | 1 | 0.00 (D) | 0.989 (Pro D) | 0.633 | 5/Yes |
| 14160 | R172G | 125 | EC | -2 | 0.00 (D) | 0.993 (Pro D) | 0.442 | 5/Yes |
| 14160 | R172W | 101 | EC | -3 | 0.00 (D) | 0.999 (Pro D) | 0.476 | 5/Yes |
| Complex III |  |  |  |  |  |  |  |  |
| MT-CYB |  |  |  |  |  |  |  |  |
| 14958 | R71P | 103 | EC | -2 | 0.00 (D) | 0.788 (Pos D) | 0.86 | 6/Yes |
| 15240 | W165Ter | NA | EC | NA | NA | NA | NA | NA/Yes |
| 15458 | S238P | 74 | EU | -1 | 0.11 (T) | 0.001 (B) | 0.431 | 2/No |
| 15522 | A259V | 64 | EC | -2 | 0.00 (D) | 0.365 (Pos D) | 0.64 | 6/Yes |
| 15596 | I284V | 29 | EU | 1 | 1.00 (T) | 0.022 (B) | 0.402 | 0/No |
| 15617 | V291I | 29 | EC | 3 | 0.00 (D) | 0.010 (B) | 0.825 | 3/Yes |
| 15651 | A302V | 64 | EU | -2 | 0.68 (T) | 0.141 (B) | 0.493 | 2/No |
| 15662 | V306I | 29 | EU | 3 | 0.48 (T) | 0.001 (B) | 0.636 | 1/No |
| 15690 | M315T | 81 | EC | -1 | 0.00 (D) | 0.006 (B) | 0.475 | 4/Yes |
| 15773 | V343M | 21 | EC | 1 | 0.00 (D) | 0.085 (B) | 0.629 | 3/Yes |
| 15851 | V369I | 29 | EU | 3 | 0.78 (T) | 0.000 (B) | 0.24 | 0/No |
| Complex IV |  |  |  |  |  |  |  |  |
| MT-CO1 |  |  |  |  |  |  |  |  |
| 5979 | A26T | 58 | EC | -1 | NA | 0.162 (B) | 0.807 | 4/Yes |
| 6160 | M86T | 81 | EC | -1 | NA | 0.102 (B) | 0.694 | 4/Yes |
| 6268 | A122V | 64 | EC | -2 | NA | 0.001 (B) | 0.668 | 4/Yes |
| 6366 | I155V | 29 | EC | 1 | NA | 0.000 (B) | 0.277 | 1/No |
| 6384 | A161T | 58 | EC | -1 | NA | 0.468 (Pos D) | 0.678 | 5/Yes |
| 6513 | A204T | 58 | EC | -1 | NA | 0.310 (Pos D) | 0.683 | 5/Yes |
| 6787 | V295A | 64 | EC | 0 | NA | 0.547 (Pos D) | 0.71 | 4/Yes |
| 7062 | F387K | 102 | EC | -3 | NA | 0.343 (Pos D) | 0.754 | 5/Yes |
| 7113 | T404A | 58 | EC | -1 | NA | 0.032 (B) | 0.36 | 3/Yes |
| 7392 | G497Ter | NA | EC | NA | NA | NA | NA | NA/Yes |
| 7419 | E506K | 56 | EC | 1 | NA | 0.007 (B) | 0.375 | 2/No |
| MT-CO2 |  |  |  |  |  |  |  |  |
| 7710 | L42P | 98 | EU | -3 | 0.00 (D) | 0.966 (Pro D) | 0.769 | 5/Yes |
| 7830 | R82H | 29 | EC | 0 | 0.00 (D) | 0.934 (Pro D) | 0.704 | 4/Yes |
| 7853 | A90V | 29 | EU | -2 | 0.23 (T) | 0.019 (B) | 0.433 | 1/No |
| 7925 | G114S | 56 | EU | 0 | 0.28 (T) | 0.596 (Pos D) | 0.3 | 2/No |
| MT-CO3 |  |  |  |  |  |  |  |  |
| 9306 | W34R | 101 | EC | -3 | 0.00 (D) | 0.814 (Pos D) | 0.883 | 6/Yes |
| 9378 | W58R | 101 | EC | -3 | 0.00 (D) | 0.585 (Pos D) | 0.899 | 6/Yes |
| 9396 | E64K | 56 | EC | 1 | 0.00 (D) | 0.997 (Pro D) | 0.86 | 5/Yes |
| 9466 | I87T | 89 | EC | -2 | 0.00 (D) | 0.714 (Pos D) | 0.763 | 6/Yes |
| 9531 | T109P | 58 | EC | 1 | 0.00 (D) | 0.810 (Pos D) | 0.576 | 5/Yes |
| 9547 | G114E | 98 | EC | -2 | 0.00 (D) | 0.976 (Pro D) | 0.581 | 6/Yes |
| 9655 | S150D | 65 | EC | 0 | 0.00 (D) | 0.698 (Pos D) | 0.821 | 5/Yes |
| 9774 | D190N | 23 | EC | 1 | 0.00 (D) | 0.748 (Pos D) | 0.848 | 4/Yes |
| 9966 | V254I | 29 | EC | 3 | 0.17 (T) | 0.000 (B) | 0.197 | 1/No |
| Complex V |  |  |  |  |  |  |  |  |
| MT-ATP8 |  |  |  |  |  |  |  |  |
| 8448 | M28T | 81 | EU | -1 | 0.17 (T) | 0.000 (B) | 0.234 | 2/No |
| 8460 | N32S | 46 | EU | 1 | 0.47 (T) | 0.021 (B) | 0.316 | 0/No |
| MT-ATP6 |  |  |  |  |  |  |  |  |
| 8557 | T11A | 58 | EU | -1 | 1.00 (T) | 0.000 (B) | 0.147 | 2/No |
| 8594 | I23T | 89 | EC | -2 | 0.00 (D) | 0.062 (B) | 0.535 | 5/Yes |
| 8654 | I43T | 89 | EU | -2 | 0.03 (D) | 0.062 (B) | 0.35 | 3/Yes |
| 8701 | T59A | 58 | EU | -1 | 0.58 (T) | 0.002 (B) | 0.086 | 2/No |
| 8743 | V73M | 21 | EU | 1 | 1.00 (T) | 0.000 (B) | 0.777 | 1/No |
| 8881 | S119P | 74 | EU | -1 | 0.18 (T) | 0.034 (B) | 0.372 | 2/No |
| 9205 | Ter-Q | NA | EC | NA | NA | NA | NA | NA/Yes |

^a^ Grantham values range from 5 to 215, in which low values indicate chemical similarity and high values indicate radical differences (Grantham, 1974) ( the cutoff value is 50). EC/EU indicates classification of nonsynonymous variants as evolutionarily conserved or evolutionarily unconserved based on sequence alignments with ten mammalian orthologs in mtSAP Evaluation of the GiiB-JST mtSNP (mitochondrial single nucleotide polymorphism) database. BLOSUM62 values range from 4 to 3, where negative values indicate less acceptable and zero and positive values indicate more acceptable substitutions. SIFT predicts whether an amino acid substitution affects protein function based on sequence homology and the physical properties of amino acids. SIFT values range from 0 to 1, where values close to 0 represent less tolerated and those near 1 represent more tolerated substitutions. When the value <0.05, program consider the substitution is deleterious. The PolyPhen-2 values range from 0 to 1, where values close to 0 represent substitution of amino acid close to benign and those near 1 represent possibly or probably damage for protein function. MutPred score represent the probability that the amino acid substitution is deleterious/disease-associated and scores > 0.5 are referred to as actionable hypotheses.

Abbreviations: SAP, single amino acid polymorphism; SIFT, sorting intolerant from tolerant; PolyPhen, polymorphism phenotyping; NA, not available; (T), Substitution of amino acid is predicted to be Tolerated; (D), Substitution of amino acid is predicted to be deleterious; (B), Benign; (Pos D), Possibly Damaging; (Pro D), Probably Damaging.
