# Supplementary material for: Clinical Significance in Oral Cavity Squamous Cell Carcinoma of Pathogenic Somatic Mitochondrial Mutations
Source: PLoS One. 2013 Jun 14;8(6):e65578. doi: 10.1371/journal.pone.0065578 (PMC3683038; doi:10.1371/journal.pone.0065578)
Supplement: Table S2 — Summary of 181 mutation positions in protein-coding genes. (DOC) [file pone.0065578.s002.doc]

Table S2. Summary of 181 mutation positions in protein-coding genes.

| Nucleotide position | Complex/Gene | rCRS | Mutation | Amino acid change | Frequency | Codon | Position | Pathogenic Prediction | Disease-Associations reported^＊^ |
| --- | --- | --- | --- | --- | --- | --- | --- | --- | --- |
| . | Complex I |  |  |  |  |  |  |  |  |
| 3337 | MT-ND1 | G | G→G/A | Val→Met | 1 | 11 | 1 | No | Polymorphic mutation (A allele, 5/2704)^a^ |
| 3380 | MT-ND1 | G | G→G/A | Arg→Gln | 2 | 25 | 2 | Yes | MELAS (A allele, 1/2704)^a^ |
| 3394 | MT-ND1 | T | T→T/C | Tyr→His | 1 | 30 | 1 | Yes | Polymorphic mutation; LHON/ NIDDM/CPT deficiency, acute leukemia platelets, leukocytes & bone marrow (C allele, 39/2704)^a^ |
| 3398 | MT-ND1 | T | T→C | Met→Thr | 1 | 31 | 2 | Yes | Polymorphic mutation; DMDF+HCM / GDM / possibly LVNC-associated (C allele, 5/2704)^a^ |
| 3460 | MT-ND1 | G | G→A | Ala→Thr | 1 | 52 | 1 | Yes | LHON (A allele, 2/2704)^a^ |
| 3528 | MT-ND1 | C | C→C/T | syn | 1 | 74 | 3 | - | NR (T allele,1/2704)^a^ |
| 3566-3571 | MT-ND1 | C6 | C6→C6-7 | Frame shift stop | 1 | 87-89 |  | Yes | Colorectal tumor, Oncocytoma |
| 3594 | MT-ND1 | C | C→C/T | syn | 1 | 96 | 3 | - | Polymorphic mutation; thyroid tumor; Haplogroup L1 L2 L0 +3592h Hpa1(T allele, 147/2704)^a^ |
| 3599 | MT-ND1 | T | T→C | Leu→Pro | 1 | 98 | 2 | Yes | NR |
| 3733 | MT-ND1 | G | G→G/A | Glu→Lys | 1 | 143 | 1 | Yes | LHON |
| 3781 | MT-ND1 | T | T→T/C | Ser→Pro | 1 | 159 | 1 | Yes | NR |
| 3980 | MT-ND1 | T | T→T/C | Met→Thr | 1 | 225 | 2 | Yes | NR |
| 4066 | MT-ND1 | C | C→C/T | Leu→Phe | 1 | 254 | 1 | Yes | NR |
| 4071 | MT-ND1 | C | T→C | syn | 1 | 255 | 3 | - | Polymorphic mutation (T allele, 47/2704)^a^ |
| 4071 | MT-ND1 | C | T→T/C | syn | 1 |  |  |  |  |
| 4106 | MT-ND1 | C | C→C/T | Thr→Ile | 1 | 267 | 2 | Yes | De novo mutation in general population |
| 4148 | MT-ND1 | G | G→G/A | Arg→His | 1 | 281 | 2 | Yes | Thyroid cancer |
| 4164 | MT-ND1 | A | A→G | syn | 1 | 286 | 3 | - | Polymorphic mutation (G allele, 38/2704)^a^ |
| 4164 | MT-ND1 | A | G→G/A | syn | 1 |  |  |  |  |
| 4170 | MT-ND1 | C | C→C/T | syn | 1 | 288 | 3 | - | Polymorphic mutation (T allele, 1/2704)^a^ |
| 4232 | MT-ND1 | T | T/C→T | Thr→Ile | 1 | 309 | 2 | Yes | Polymorphic mutation (C allele, 15/2704)^a^ |
| 4491 | MT-ND2 | G | G→A | Val→Ile | 1 | 8 | 1 | No | Polymorphic mutation (A allele, 18/2704)^a^ |
| 4491 | MT-ND2 | G | G→G/A | Val→Ile | 1 |  |  |  |  |
| 4665 | MT-ND2 | G | G→A | Ala→Thr | 1 | 66 | 1 | Yes | Breast cancer |
| 4820 | MT-ND2 | G | A→A/G | syn | 1 | 117 | 3 | - | Polymorphic mutation (A allele, 45/2704)^a^ |
| 4883 | MT-ND2 | C | T→T/C | syn | 1 | 138 | 3 | - | Polymorphic mutation (A allele, 18/2704)^a^ |
| 5007 | MT-ND2 | G | G→G/A | Ala→Thr | 1 | 180 | 1 | Yes | NR |
| 5093 | MT-ND2 | T | T→T/C | syn | 1 | 208 | 3 | - | Polymorphic mutation (C allele, 4/2704)^a^ |
| 5104 | MT-ND2 | C | C→C/T | Thr→Ile | 1 | 212 | 2 | Yes | NR |
| 5140 | MT-ND2 | G | G→G/A | Ser→Asn | 1 | 224 | 2 | No | Polymorphic mutation; Colonic crypts |
| 5147 | MT-ND2 | G | G→G/A | syn | 1 | 226 | 3 | - | Polymorphic mutation (A allele, 135/2704)^a^ |
| 5178 | MT-ND2 | C | A→A/C | Met→Leu | 1 | 237 | 1 | No | Polymorphic mutation; Longevity/ Extraversion/ MI protection; L-M Haplogroup D -5176a Alu1 (A allele, 299/2704)^a^ |
| 5231 | MT-ND2 | G | G/A→A | syn | 1 | 254 | 3 | - | Polymorphic mutation; Endometrium control tissue (A allele, 70/2704)^a^ |
| 5231 | MT-ND2 | G | G→G/A | syn | 1 |  |  |  |  |
| 5351 | MT-ND2 | A | G/A→A | syn | 2 | 294 | 3 | - | Polymorphic mutation (G allele, 40/2704)^a^ |
| 5351 | MT-ND2 | A | G→G/A | syn | 1 |  |  |  |  |
| 5417 | MT-ND2 | G | G→G/A | syn | 1 | 316 | 3 | - | Polymorphic mutation (G allele, 63/2704)^a^ |
| 5460 | MT-ND2 | G | A→A/G | Thr→Ala | 1 | 331 | 1 | No | AD/PD |
| 5465 | MT-ND2 | T | T→C | syn | 1 | 332 | 3 | - | Polymorphic mutation (C allele, 47/2704)^a^ |
| 5471 | MT-ND2 | G | G→G/A | syn | 1 | 334 | 3 | - | Polymorphic mutation (A allele, 23/2704)^a^ |
| 5474 | MT-ND2 | A | G/A→A | syn | 2 | 335 | 3 | - | Polymorphic mutation (G allele, 2/2704)^a^ |
| 5498 | MT-ND2 | A | G/A→A | syn | 2 | 343 | 3 |  | Polymorphic mutation (G allele, 4/2704)^a^ |
| 10068 | MT-ND3 | G | G→G/A | Ala→Thr | 1 | 4 | 1 | No | NR |
| 10158 | MT-ND3 | T | T→T/C | Ser→Pro | 1 | 34 | 1 | Yes | Leigh Disease/ MNGIE tissues |
| 10172 | MT-ND3 | G | A/G→A | syn | 1 | 38 | 3 | - | Polymorphic mutation (A allele, 11/2704)^a^ |
| 10243 | MT-ND3 | T | T→T/C | Phe→Ser | 1 | 62 | 2 | Yes | NR |
| 10245 | MT-ND3 | T | T→T/C | syn | 1 | 63 | 1 | - | Polymorphic mutation (C allele, 9/2704)^a^ |
| 10371 | MT-ND3 | G | G→G/A | Glu→Lys | 1 | 105 | 1 | Yes | NR |
| 10398 | MT-ND3 | A | A→A/G | Thr→Ala | 1 | 114 | 1 | No | Polymorphic mutation; PD protective factor/ longevity/ altered cell pH; thyroid tumor (G allele, 1242/2704)^a^ |
| 10400 | MT-ND3 | C | C→C/T | syn | 1 | 114 | 3 | - | Polymorphic mutation; supergroup M (T allele, 724/2704)^a^ |
| 10579 | MT-ND4L | T | T→T/C | Met→Thr | 1 | 37 | 2 | Yes | Renal tumor |
| 10873 | MT-ND4 | T | T→T/C | syn | 1 | 38 | 3 | - | Polymorphic mutation (C allele, 943/2704)^a^ |
| 10873 | MT-ND4 | T | C→C/T | syn | 1 |  |  |  |  |
| 11032 | MT-ND4 | A | A→A/G | syn | 1 | 91 | 3 | - | NR |
| 11040 | MT-ND4 | T | T→T/C | Leu→Pro | 1 | 94 | 2 | Yes | NR |
| 11055 | MT-ND4 | T | T→T/C | Leu→Pro | 1 | 99 | 2 | Yes | NR |
| 11148 | MT-ND4 | T | T→C | Leu→Ser | 1 | 130 | 2 | Yes | NR |
| 11215 | MT-ND4 | C | T→T/C | syn | 1 | 152 | 3 | - | Polymorphic mutation (T allele, 36/2704)^a^ |
| 11394 | MT-ND4 | T | T→T/C | Leu→Pro | 1 | 212 | 2 | Yes | Polymorphic mutation |
| 11406 | MT-ND4 | T | T→T/C | Leu→Pro | 1 | 216 | 2 | Yes | NR |
| 11432 | MT-ND4 | A | A/G→G | Ile→Val | 1 | 225 | 1 | Yes | De novo mutation in general population |
| 11711 | MT-ND4 | G | G→G/A | Ala→Thr | 2 | 318 | 1 | Yes | De novo mutation in general population |
| 11867-11872 | MT-ND4 | C6 | C6→C6-7 | Frame shift stop | 1 | 370-371 |  | Yes | Pituitary oncocytoma |
| 11982 | MT-ND4 | T | T→C | Leu→Pro | 1 | 408 | 2 | Yes | NR |
| 12008 | MT-ND4 | G | G→G/A | Gly→Ser | 1 | 417 | 1 | Yes | NR |
| 12026 | MT-ND4 | A | A→A/G | Ile→Val | 1 | 423 | 1 | No | DM (G allele, 24/2704)^a^ |
| 12030 | MT-ND4 | A | A→A/G | Asn→Ser | 1 | 424 | 2 | No | Polymorphic mutation (G allele, 3/2704)^a^ |
| 12082 | MT-ND4 | A | A→A/G | syn | 1 | 441 | 3 | - | De novo mutation in general population (G allele, 1/2704)^a^ |
| 12088 | MT-ND4 | C | C→C/T | syn | 1 | 443 | 3 | - | NR (T allele, 2/2704)^a^ |
| 12127 | MT-ND4 | G | G→G/A | syn | 1 | 456 | 3 | - | Polymorphic mutation (A allele, 5/2704)^a^ |
| 12128 | MT-ND4 | T | T→T/C | Phe→Leu | 1 | 457 | 1 | No | NR |
| 12358 | MT-ND5 | A | A→A/G | Thr→Ala | 1 | 8 | 1 | No | Polymorphic mutation (G allele, 60/2704)^a^ |
| 12372 | MT-ND5 | G | G→G/A | syn | 1 | 12 | 3 | - | Polymorphic mutation; Altered brain pH; prostate tumor (A allele, 390/2704)^a^ |
| 12372 | MT-ND5 | G | A→A/G | syn | 1 |  |  |  |  |
| 12398 | MT-ND5 | C | T→T/C | Ile→Thr | 1 | 21 | 2 | No | NR |
| 12399 | MT-ND5 | C | C→C/T | syn | 1 | 21 | 3 | - | Polymorphic mutation |
| 12418-12425 | MT-ND5 | A8 | A8→A8-9 | Frame shift stop | 4 | 28-30 |  | Yes | Colorectal tumor |
| 12418-12425 | MT-ND5 | A8 | A8→A7-8 | Frame shift stop | 1 | 28-30 |  |  | Pituitary oncocytoma |
| 12508 | MT-ND5 | G | G→G/A | Asp→Asn | 1 | 58 | 1 | No | De novo mutation in general population |
| 12547 | MT-ND5 | A | A→A/G | Thr→Ala | 1 | 71 | 1 | Yes | NR |
| 12561 | MT-ND5 | G | G/A→A | syn | 1 | 75 | 3 | - | Polymorphic mutation; pancreatic cancer cell line (A allele, 4/2704)^a^ |
| 12572 | MT-ND5 | G | G→A | Ser→Asn | 1 | 79 | 2 | Yes | NR |
| 12590 | MT-ND5 | T | T→T/C | Phe→Ser | 1 | 85 | 2 | Yes | NR |
| 12705 | MT-ND5 | C | C→C/T | syn | 1 | 123 | 3 | - | Polymorphic mutation; prostate tumor (T allele, 1223/2704)^a^ |
| 12705 | MT-ND5 | C | T→T/C | syn | 2 |  |  |  |  |
| 12714 | MT-ND5 | T | T→T/C | syn | 1 | 126 | 3 | - | Polymorphic mutation (C allele, 13/2704)^a^ |
| 12727 | MT-ND5 | T | T→C | syn | 1 | 131 | 1 | - | Polymorphic mutation (C allele, 2/2704)^a^ |
| 12736 | MT-ND5 | G | G→G/A | Ala→Thr | 1 | 134 | 1 | Yes | NR |
| 12748 | MT-ND5 | T | T→T/C | Phe→Leu | 1 | 138 | 1 | No | NR |
| 12822 | MT-ND5 | A | A→C | syn | 1 | 162 | 3 | - | NR |
| 13154 | MT-ND5 | T | T→T/C | Ile→Thr | 1 | 273 | 2 | Yes | De novo mutation in general population |
| 13191 | MT-ND5 | T | T→C | syn | 1 | 285 | 3 | - | De novo mutation in general population (C allele, 2/2704)^a^ |
| 13231-13237 | MT-ND5 | A7 | A7→A7-8 | Frame shift stop | 1 | 299-301 |  | Yes | NR |
| 13269 | MT-ND5 | A | A→A/G | syn | 1 | 311 | 3 | - | Polymorphic mutation (G allele, 6/2704)^a^ |
| 13406 | MT-ND5 | G | G→G/A | Arg→Glu | 1 | 357 | 2 | Yes | NR |
| 13443 | MT-ND5 | T | C→C/T | syn | 1 | 369 | 3 | - | Polymorphic mutation (C allele, 2/2704)^a^ |
| 13590 | MT-ND5 | G | A→A/G | syn | 1 | 418 | 3 | - | Polymorphic mutation (A allele, 110/2704)^a^ |
| 13610 | MT-ND5 | G | G→G/A | Arg→Gln | 1 | 425 | 2 | Yes | NR |
| 13753 | MT-ND5 | T | T→T/C | Ser→Pro | 1 | 473 | 1 | No | Polymorphic mutation (C allele, 1/2704)^a^ |
| 13904 | MT-ND5 | C | C→T | Ser→Pro | 1 | 523 | 2 | Yes | NR |
| 13908 | MT-ND5 | C | C→C/T | syn | 1 | 524 | 3 | - | NR |
| 13928 | MT-ND5 | G | G→G/C | Ser→Thr | 1 | 531 | 2 | No | Polymorphic mutation (C allele, 90/2704)^a^ |
| 13980 | MT-ND5 | G | G→G/A | syn | 1 | 548 | 3 | - | Polymorphic mutation (A allele, 10/2704)^a^ |
| 14036 | MT-ND5 | C | C→C/T | Ser→Leu | 1 | 567 | 2 | Yes | NR |
| 14045 | MT-ND5 | A | A→A/G | Gln→Arg | 1 | 570 | 2 | Yes | NR |
| 14133 | MT-ND5 | A | A→A/G | syn | 1 | 599 | 3 | - | Polymorphic mutation (G allele, 18/2704)^a^ |
| 14133 | MT-ND5 | A | G/A→A | syn | 2 |  |  |  |  |
| 14160 | MT-ND6 | G | G→C | Arg→Gly | 1 | 172 | 1 | Yes | NR |
| 14160 | MT-ND6 | G | G→G/A | Arg→Trp | 1 | 172 | 1 | Yes | De novo mutation in general population |
| 14169 | MT-ND6 | C | C→C/T | Glu→Lys | 1 | 169 | 1 | Yes | De novo mutation in general population |
| 14208 | MT-ND6 | T | C/T→T/C | Ala→Thr | 1 | 156 | 1 | Yes | De novo mutation in general population |
| 14226 | MT-ND6 | G | G→A | Arg→Cys | 1 | 150 | 1 | Yes | De novo mutation in general population |
| 14308 | MT-ND6 | C | T→T/C | syn | 1 | 122 | 3 | - | Polymorphic mutation (C allele, 39/2704)^a^ |
| 14587 | MT-ND6 | A | G→G/A | syn | 1 | 29 | 3 | - | Polymorphic mutation (G allele, 4/2704)^a^ |
| 14615 | MT-ND6 | G | G→G/GG | Frame shift stop | 1 | 20 | 2 | Yes | NR |
| 14668 | MT-ND6 | C | T→T/C | syn | 1 | 2 | 3 | - | Polymorphic mutation; MDD-associated (T allele, 272/2704)^a^ |
|  |  |  |  |  |  |  |  |  |  |
|  | Complex III |  |  |  |  |  |  |  |  |
| 14770 | MT-CYB | C | C→C/T | syn | 1 | 8 | 3 | - | Polymorphic mutation (T allele, 3/2704)^a^ |
| 14783 | MT-CYB | T | T→T/C | syn | 1 | 13 | 1 | - | Polymorphic mutation (C allele, 720/2704)^a^ |
| 14783 | MT-CYB | T | C→C/T | syn | 1 |  |  |  |  |
| 14857 | MT-CYB | T | T→T/C | syn | 1 | 37 | 3 | - | Polymorphic mutation (C allele, 1/2704)^a^ |
| 14958 | MT-CYB | G | G→G/C | Arg→Pro | 1 | 71 | 2 | Yes | NR |
| 15010 | MT-CYB | A | A→A/G | syn | 1 | 88 | 3 | - | Polymorphic mutation (G allele, 1/2704)^a^ |
| 15043 | MT-CYB | G | G→G/A | syn | 1 | 99 | 3 | - | Polymorphic mutation; MDD-associated (A allele, 777/2704)^a^ |
| 15043 | MT-CYB | G | G/A→A/G | syn | 1 |  |  |  |  |
| 15043 | MT-CYB | G | A→A/G | syn | 1 |  |  |  |  |
| 15046 | MT-CYB | A | G/A→A/G | syn | 1 | 100 | 3 | - | De novo mutation in general population (G allele, 1/2704)^a^ |
| 15240 | MT-CYB | G | G→G/A | Trp→Ter | 1 | 165 | 2 | Yes | NR |
| 15244 | MT-CYB | A | A/G→G | syn | 1 | 166 | 3 | - | Polymorphic mutation (G allele, 27/2704)^a^ |
| 15301 | MT-CYB | G | G→G/A | syn | 1 | 185 | 3 | - | Polymorphic mutation; Tumor (A allele, 867/2704)^a^ |
| 15301 | MT-CYB | G | G/A→A | syn | 2 |  |  |  |  |
| 15301 | MT-CYB | G | A→A/G | syn | 1 |  |  |  |  |
| 15301 | MT-CYB | G | G/A→G | syn | 1 |  |  |  |  |
| 15458 | MT-CYB | T | T→T/C | Ser→Pro | 1 | 238 | 1 | No | Polymorphic mutation (C allele, 2/2704)^a^ |
| 15463 | MT-CYB | A | A→A/G | syn | 1 | 239 | 3 | - | Polymorphic mutation |
| 15508 | MT-CYB | C | T/C→C | syn | 2 | 254 | 3 | - | Polymorphic mutation (T allele, 29/2704)^a^ |
| 15522 | MT-CYB | C | C→C/T | Ala→Val | 1 | 259 | 2 | Yes | NR |
| 15535 | MT-CYB | C | T→T/C | syn | 1 | 263 | 3 | - | Polymorphic mutation (T allele, 48/2704)^a^ |
| 15596 | MT-CYB | G | A→A/G | Ile→Val | 1 | 284 | 1 | No | De novo mutation in general population |
| 15617 | MT-CYB | G | G→G/A | Val→Ile | 1 | 291 | 1 | Yes | Polymorphic mutation |
| 15651 | MT-CYB | C | C→C/T | Ala→Val | 1 | 302 | 2 | No | Polymorphic mutation |
| 15662 | MT-CYB | A | G/A→A | Val→Ile | 2 | 306 | 1 | No | Polymorphic mutation; Complex mitochondriopathy-associated (G allele, 28/2704)^a^ |
| 15690 | MT-CYB | T | T→T/C | Met→Thr | 1 | 315 | 2 | Yes | NR |
| 15773 | MT-CYB | G | G→G/A | Val→Met | 1 | 343 | 1 | Yes | Polymorphic mutation; LHON (A allele, 5/2704)^a^ |
| 15851 | MT-CYB | A | G/A→A | Val→Ile | 2 | 369 | 1 | No | Polymorphic mutation (G allele, 28/2704)^a^ |
|  |  |  |  |  |  |  |  |  |  |
|  | Complex IV |  |  |  |  |  |  |  |  |
| 5979 | MT-CO1 | G | G→A | Ala→Thr | 1 | 26 | 1 | Yes | Polymorphic mutation (A allele, 1/2704)^a^ |
| 6160 | MT-CO1 | T | T→C | Met→Thr | 1 | 86 | 2 | Yes | NR |
| 6268 | MT-CO1 | C | C→C/T | Ala→Val | 1 | 122 | 2 | Yes | LHON; De novo mutation in general population |
| 6366 | MT-CO1 | G | A/G→G | Ile→Val | 1 | 155 | 1 | No | Polymorphic mutation (A allele, 9/2704)^a^ |
| 6384 | MT-CO1 | G | G→A | Ala→Thr | 1 | 161 | 1 | Yes | Prostate tumor |
| 6455 | MT-CO1 | C | T→T/C | syn | 1 | 184 | 3 | - | Polymorphic mutation (T allele, 96/2704)^a^ |
| 6513 | MT-CO1 | G | G→G/A | Ala→Thr | 1 | 204 | 1 | Yes | Esophageal cancer |
| 6572 | MT-CO1 | C | C→C/T | syn | 1 | 223 | 3 | - | NR |
| 6620 | MT-CO1 | T | T/C→C | syn | 1 | 239 | 3 | - | Polymorphic mutation (C allele, 12/2704)^a^ |
| 6680 | MT-CO1 | T | C→C/T | syn | 1 | 259 | 3 | - | Polymorphic mutation (C allele, 98/2704)^a^ |
| 6686 | MT-CO1 | C | C→C/T | syn | 1 | 281 | 3 | - | NR |
| 6787 | MT-CO1 | T | T→T/C | Val→Ala | 1 | 295 | 2 | Yes | Hepatocellular tumor |
| 6932 | MT-CO1 | A | A→A/G | syn | 1 | 343 | 3 | - | De novo mutation in general population (G allele, 1/2704)^a^ |
| 7055 | MT-CO1 | A | A→A/G | syn | 1 | 384 | 3 | - | Polymorphic mutation; MNGIE fibroblasts (G allele, 52/2704)^a^ |
| 7062 | MT-CO1 | T | T→T/C | Phe→Lys | 1 | 387 | 1 | Yes | NR |
| 7113 | MT-CO1 | A | A→A/G | Thr→Ala | 1 | 404 | 1 | Yes | De novo mutation in general population (G allele, 1/2704)^a^ |
| 7392 | MT-CO1 | G | G→G/A | Gly→Ter | 1 | 497 | 1 | Yes | NR |
| 7419 | MT-CO1 | G | G→G/A | Glu→Lys | 1 | 506 | 1 | No | De novo mutation in general population (A allele, 2/2704)^a^ |
| 7684 | MT-CO2 | T | C→C/T | syn | 1 | 33 | 3 | - | Polymorphic mutation (C allele, 98/2704)^a^ |
| 7710 | MT-CO2 | T | T→C | Leu→Pro | 1 | 42 | 2 | Yes | Recurrent pregnancy loss |
| 7830 | MT-CO2 | G | G→G/A | Arg→His | 1 | 82 | 2 | Yes | De novo mutation in general population (A allele, 4/2704)^a^ |
| 7853 | MT-CO2 | G | A→A/G | Ala→Val | 1 | 90 | 1 | No | Polymorphic mutation (A allele, 45/2704)^a^ |
| 7925 | MT-CO2 | G | G→G/A | Gly→Ser | 2 | 114 | 1 | No | Papillary thyroid carcinoma |
| 8028-8032 | MT-CO2 | C5 | C5→C5-6 | Frame shift stop | 1 | 148-149 |  | Yes | NR |
| 9242 | MT-CO3 | A | A→A/G | syn | 1 | 12 | 3 | - | Polymorphic mutation (G allele, 16/2704)^a^ |
| 9306 | MT-CO3 | T | T→T/C | Trp→Arg | 1 | 34 | 1 | Yes | NR |
| 9378 | MT-CO3 | T | T→T/C | Trp→Arg | 1 | 58 | 1 | Yes | Head and neck cancer |
| 9386 | MT-CO3 | T | T→T/C | syn | 1 | 60 | 3 | - | Polymorphic mutation (C allele, 5/2704)^a^ |
| 9396 | MT-CO3 | G | G→G/A | Glu→Lys | 1 | 64 | 1 | Yes | NR |
| 9466 | MT-CO3 | T | T→T/C | Ile→Thr | 1 | 87 | 2 | Yes | NR |
| 9530 | MT-CO3 | T | T/C→C | syn | 1 | 108 | 3 | - | De novo mutation in general population (C allele, 2/2704)^a^ |
| 9531 | MT-CO3 | A | A→A/C | Thr→Pro | 1 | 109 | 1 | Yes | NR |
| 9532-9537 | MT-CO3 | C6 | C6→C6-7 | Frame shift stop | 3 | 109-111 | - | Yes | Leigh Syndrome; colonic crypts |
| 9540 | MT-CO3 | T | T→T/C | syn | 2 | 112 | 1 | - | Polymorphic mutation; Tumor (C allele, 944/2704)^a^ |
| 9540 | MT-CO3 | T | C/T→T | syn | 2 |  |  |  |  |
| 9545 | MT-CO3 | A | G/A→A | syn | 1 | 113 | 3 | - | Polymorphic mutation (G allele, 54/2704)^a^ |
| 9547 | MT-CO3 | G | G→A | Gly→Glu | 1 | 114 | 2 | Yes | Cloned platelet mtDNA |
| 9638 | MT-CO3 | C | C→C/T | syn | 1 | 144 | 3 | - | NR |
| 9655 | MT-CO3 | G | G→G/A | Ser→Asp | 1 | 150 | 2 | Yes | Polymorphic mutation; thyroid tumor |
| 9774 | MT-CO3 | G | G→G/A | Asp→Asn | 1 | 190 | 1 | Yes | Colonic crypts |
| 9966 | MT-CO3 | G | G→G/A | Val→Ile | 1 | 254 | 1 | No | Polymorphic mutation (A allele, 16/2704)^a^ |
| 9986 | MT-CO3 | G | G→G/A | syn | 1 | 260 | 3 | - | Gastric cancer (A allele, 3/2704)^a^ |
|  |  |  |  |  |  |  |  |  |  |
|  | Complex V |  |  |  |  |  |  |  |  |
| 8389 | MT-ATP8 | A | A/G→G | syn | 1 | 8 | 3 | - | Polymorphic mutation (G allele, 4/2704)^a^ |
| 8406-8410 | MT-ATP8 | C5 | C5→C5-6 | Frame shift stop | 1 | 14-15 |  | Yes | NR |
| 8448 | MT-ATP8 | T | T→T/C | Met→Thr | 1 | 28 | 2 | No | Polymorphic mutation (C allele, 5/2704)^a^ |
| 8460 | MT-ATP8 | A | A→G | Asn→Ser | 1 | 32 | 2 | No | Polymorphic mutation (G allele, 17/2704)^a^ |
| 8557 | MT-ATP6/8 | G | A→A/G | ATP6:Thr→Ala ATP8:syn | 1 | 11/64 | 1/3 | No | Polymorphic mutation; colonic crypts (A allele, 21/2704)^a^ |
| 8594 | MT-ATP6 | T | T→C | Ile→Thr | 1 | 23 | 2 | Yes | Polymorphic mutation (C allele, 11/2704)^a^ |
| 8654 | MT-ATP6 | T | T→T/C | Ile→Thr | 1 | 43 | 2 | Yes | De novo mutation in general population (C allele, 2/2704)^a^ |
| 8701 | MT-ATP6 | A | A→A/G | Thr→Ala | 1 | 59 | 1 | No | Polymorphic mutation; thyroid tumors (G allele, 933/2704)^a^ |
| 8701 | MT-ATP6 | A | G→G/A | Ala→Thr | 1 |  |  |  |  |
| 8743 | MT-ATP6 | G | G→G/A | Val→Met | 1 | 73 | 1 | No | De novo mutation in general population (A allele, 1/2704)^a^ |
| 8743 | MT-ATP6 | G | A/G→G/A | Met→Val | 1 |  |  |  |  |
| 8766 | MT-ATP6 | C | C→C/T | syn | 1 | 80 | 3 | - | De novo mutation in general population (T allele, 1/2704)^a^ |
| 8772 | MT-ATP6 | T | C/T→T | syn | 1 | 82 | 1 | - | De novo mutation in general population (C allele, 3/2704)^a^ |
| 8881 | MT-ATP6 | T | T→T/C | Ser→Pro | 1 | 119 | 1 | No | NR |
| 9205 | MT-ATP6 | T | T→T/C | Ter→Gln | 1 | stop | 1 | Yes | Polymorphic mutation |

^＊^Database reference :

Mitomap: http://mitomap.org/MITOMAP

mitoWheel: http://mitowheel.org/mitowheel.html

^a^mtDB: http://www.genpat.uu.se/mtDB/

Abbreviations: LHON, Leber hereditary optic neuropathy; MM, mitochondrial myopathy; AD, Alzeimer's disease; LIMM, lethal infantile mitochondrial myopathy; ADPD, Alzeimer's disease and parkinsons's disease; MMC, maternal myopathy and cardiomyopathy; NARP, neurogenic muscle weakness, ataxia, and retinitis pigmentosa (alternate phenotype at this locus is reported as Leigh disease); FICP, fatal infantile cardiomyopathy plus, a MELAS-associated cardiomyopathy; MELAS, mitochondrial encephalomyopathy, lactic acidosis, and stroke-like episodes; LDYT, Leber's hereditary optic neuropathy and DYsTonia; MERRF, myoclonic epilepsy and ragged red muscle fibers; MHCM, maternally inherited hypertrophic cardiomyopathy; CPEO, chronic progressive external ophthalmoplegia; KSS, Kearns Sayre syndrome; DM, diabetes mellitus; DMDF, diabetes mellitus + deaFness; CIPO, chronic intestinal pseudoobstruction with myopathy and ophthalmoplegia; DEAF, maternally inherited deafness or aminoglycoside-induced deafness; PEM, progressive encephalopathy; SNHL, sensory neural hearing loss; noncod, none coding;NR, not reported; syn, synonymous mutation.
