# Supplementary material for: Clinical Significance in Oral Cavity Squamous Cell Carcinoma of Pathogenic Somatic Mitochondrial Mutations
Source: PLoS One. 2013 Jun 14;8(6):e65578. doi: 10.1371/journal.pone.0065578 (PMC3683038; doi:10.1371/journal.pone.0065578)
Supplement: Table S3 — Combined effect of TP53 R72P polymorphism and mutation on pathogenic somatic mtDNA mutation. (DOC) [file pone.0065578.s003.doc]

Table S3. Combined effect of TP53 R72P polymorphism and mutation on pathogenic somatic mtDNA mutation

| TP53 R72P genotype/ p53 mutation | mtDNA pathogenic mutation | | |
| --- | --- | --- | --- |
|  | N (%) | OR (95% CI) | *p* |
| PP/ - (n = 45) | 6 (13.3) | 1.0 |  |
| PP/ + (n = 22) | 3 (13.6) | 1.03 (0.18-5.41) | 1.00 |
| RR+RP/ - (n = 41) | 6 (14.6) | 1.11 (0.28-4.39) | 0.86 |
| RR+RP/ + (n = 52) | 18 (34.6) | 3.44 (1.12-11.05) | 0.02 |
